# The complete chloroplast genome sequence of *Pinus bhutanica* (Pinaceae) and its phylogenetic implications

**DOI:** 10.1080/23802359.2024.2305710

**Published:** 2024-01-26

**Authors:** Shiqi Lv, Jiao Chen, Bingbing Li, Taotao Fu, Mingliang Song, Pengtao Zhang, Kang Liu, Yixuan Kou, Jing Wang

**Affiliations:** a College of Life Sciences and Engineering, Henan University of Urban Construction, Pingdingshan, China; b Xifeng Experimental Station on Soil and Water Conservation of Yellow River Conservancy Commission, Qingyang, China; c Conservation Center of Shahe National Wetland Park, Luohe, China; d Laboratory of Subtropical Biodiversity, Jiangxi Agricultural University, Nanchang, China

**Keywords:** *Pinus bhutanica*, chloroplast genome, phylogenetic tree, subsection *Strobus*, Pinaceae

## Abstract

*Pinus bhutanica* is a critically endangered conifer and occurs only in central Bhutan, northwestern Yunnan and southeastern Xizang in China. In this study, the complete chloroplast genome of *Pinus bhutanica* was first assembled based on next-generation sequencing. The genome sequence was 116,919 bp in length with an overall GC content of 38.75%. A total of 106 functional genes were detected in the genome, including 72 protein-coding genes (PCGs), 30 transfer RNA (tRNA) genes, and four ribosomal RNA (rRNA) genes. The phylogenetic tree reconstructed by 12 chloroplast genomes revealed that *P. bhutanica* is most closely related to *Pinus wangii* in subsection *Strobus* of *Pinus*.

## Introduction

*Pinus bhutanica* Grierson, D. G. Long & C. N. Page 1980 belongs to the subsection *Strobus* in *Pinus* (Gernandt et al. 2005). This species has an extremely narrow distribution and occurs only in central Bhutan, northwestern Yunnan, and southeastern Xizang in China (Farjon 1998). Although phylogenetic analyses have reconstructed a fully resolved phylogeny of the species in the subsection *Strobus* based on thousands of nuclear genes (Liu et al. 2022), the interspecific relationships of these species, and including *P. bhutanica*, are still unclear. The chloroplast genome is maternally inherited in coniferous trees (Mogensen 1996), making it an important resource for exploring the phylogenetic relationships of these species (Kress et al. 2005). However, a complete chloroplast genome of *P. bhutanica* is lacking. In this study, we sequenced and analyzed the complete chloroplast genome sequence of *P. bhutanica*, and inferred the phylogenetic relationships of this species in the subsection *Strobus*.

## Materials and methods

**Figure 1.**
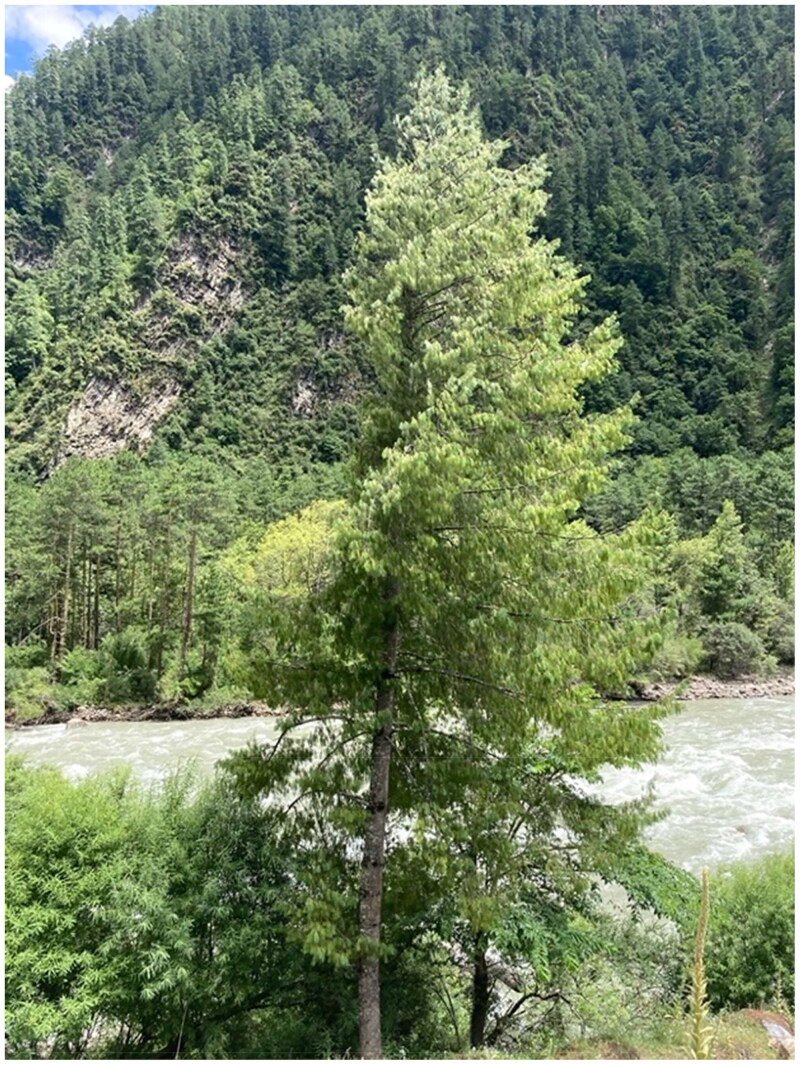
The reference image of *Pinus bhutanica* living in Linzhi, Xizang, China (geographic coordinates: 29°13′27″ N, 95°11′3″ E; photography by Yixuan Kou and Jing Wang).

Fresh leaves of *P. bhutanica* were collected from a single individual living in Linzhi, Xizang, China (geographic coordinates: 29°13′27″ N, 95°11′3″ E) (Figure 1, Figure S1). Total genomic DNA was extracted from approximately 20 mg of dried leaves using a modified CTAB procedure (Doyle and Doyle 1987). A voucher specimen (2021-PB-1A) and an associated DNA sample were stored in the Herbarium of Jiangxi Agricultural University, Nanchang, China. Whole-genome sequencing was conducted on the DNBseq platform (BGI, Shenzhen, China) with paired-end reads of 150 bp. A total of 6.4 Gb clean data (∼20 million reads) with a Q20 of 97.87% were obtained and subsequently used for chloroplast genome assembly. The assembly was performed using NOVOPlasty 4.3.1 (Dierckxsens et al. 2016), and annotation of the assembled genome was performed by Plastid Genome Annotator (PGA) (Qu et al. 2019) with default parameters and the closely related species *Pinus koraiensis* Siebold et Zuccarini (NC_0046772.2) as the reference. The detailed chloroplast genome structure was visualized using CPGView (Liu et al. 2023). To verify the accuracy of the assembly, we mapped clean reads to the assembled chloroplast genome to assess the depth of coverage using a python script (Ni et al. 2023).

The phylogenetic relationships of *P. bhutanica* in subsection *Strobus* were explored based on chloroplast genome datasets. The chloroplast genome sequences of 10 species in subsection *Strobus* were downloaded and aligned with the genome of *P. bhutanica* using MAFFT v7.310 (Katoh and Standley 2013). *Pinus krempfii* Lecomte was used as the outgroup. A maximum-likelihood (ML) tree was constructed by FastTree 2.1.11 (Price et al. 2009) with the GTR substitution model.

## Results

**Figure 2.**
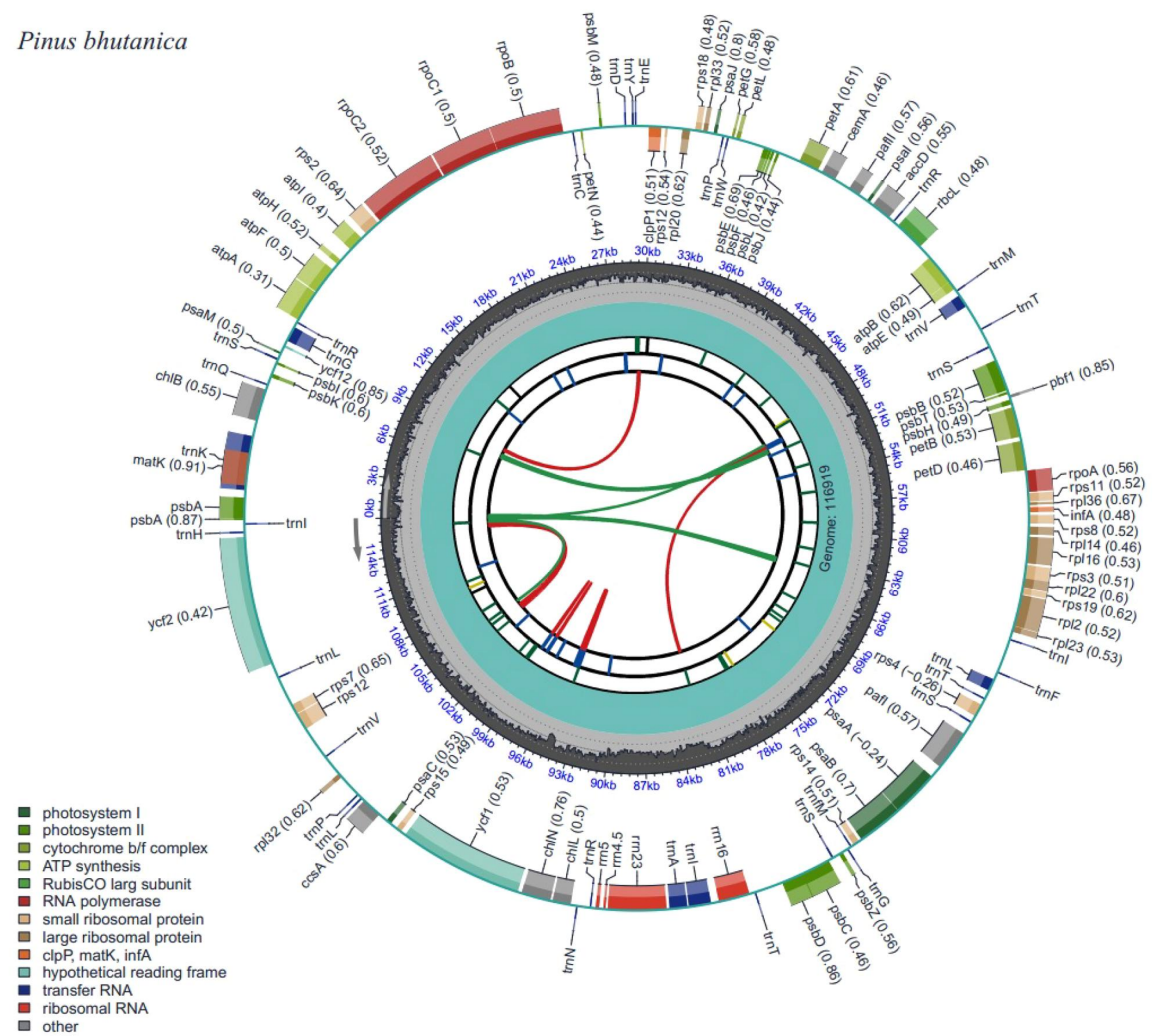
Genomic map of overall features of *Pinus bhutanica* chloroplast genome, generated by CPGView (Liu et al. 2023). The map contains six tracks by default. From the center outward, the first track shows the dispersed repeats. The dispersed repeats consist of direct and palindromic repeats, connected with red and green arcs. The second track shows the long tandem repeats as short blue bars. The third track shows the short tandem repeats or microsatellite sequences as short bars with different colors. The genome length and the GC content along the genome are shown on the fourth and fifth track, respectively. The genes are shown on the sixth track. Genes are color-coded by their functional classification.

**Figure 3.**
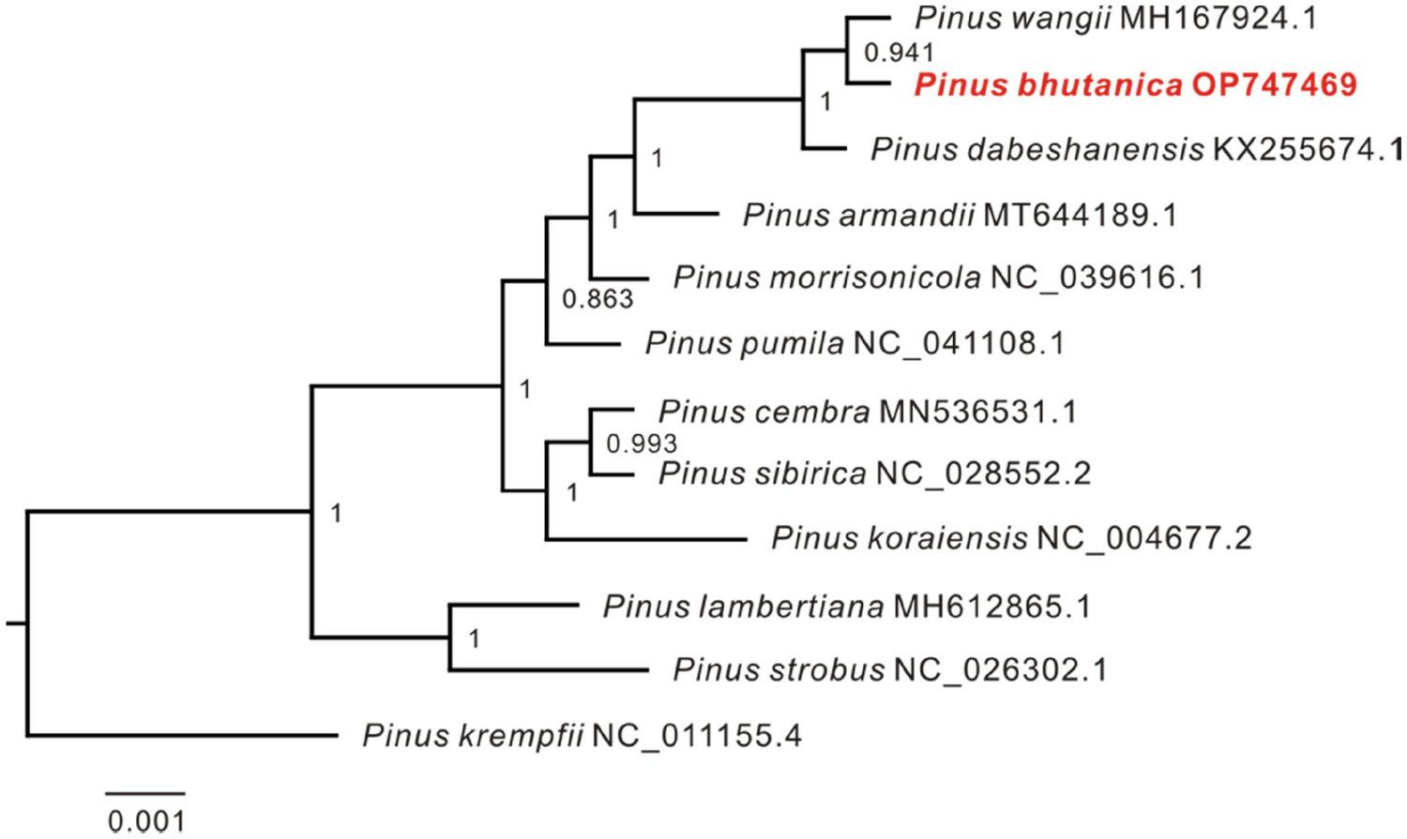
Maximum-likelihood (ML) tree inferred by FastTree with the GTR substitution model based on the complete chloroplast genome sequences of *P. bhutanica* and 10 other species in subsection *Strobus*. The numbers on the nodes represent the support values. The following sequences were used: *Pinus wangii* MH167924.1 (Yang et al. 2018), *Pinus dabeshanensis* KX255674.1 (Duan et al. 2016), *Pinus armandii* MT644189.1 (Jia et al. 2020), *Pinus morrisonicola* NC_039616.1 (Zeb et al. 2020), *Pinus pumila* NC_041108.1 (Zeb et al. 2019), *Pinus cembra* MN536531.1 (Schott et al. 2019), *Pinus sibirica* NC_028552.2 (Baturina et al. 2019), *Pinus koraiensis* NC_004677.2 (available in the GenBank of NCBI), *Pinus lambertiana* MH612865.1 (Gernandt et al. 2018), *Pinus strobus* NC_026302.1 (Zhu et al. 2016), and *Pinus krempfii* NC_011155.4 (Cronn et al. 2008).

The complete chloroplast genome of *P. bhutanica* was deposited in the GenBank database under accession number OP747469. It was circular and 116,919 bp in length with an overall GC content of 38.75% and an average coverage of 312.62× (Figure 2, Figure S2). A total of 106 functional genes were annotated in the genome, including 72 protein-coding genes (PCGs), 30 transfer RNA (tRNA) genes, and four ribosomal RNA (rRNA) genes. Most of these genes occurred as a single copy and had no introns, except that *psbA*, *trnR-ACG*, *trnS-GCU*, and *trnT-GGU* had two copies, *trnI-GAU* had three copies, and 12 genes (*atpF*, *petB*, *petD*, *rpl16*, *rpl2*, *rpoC1*, *trnA-UGC*, *trnG-UCC*, *trnI-GAU*, *trnK-UUU*, *trnL-UAA*, and *trnV-UAC*) had one intron and two genes (*rps12* and *pafI*) had two introns (Figure 2). In addition, seven cis-splicing genes (*atpF*, *rpoC1*, *petB*, *petD*, *rpl16*, *rpl2*, and *pafI*) and one trans-splicing gene (*rps12*) were detected (Figure S3). The phylogenetic analyses showed that all species in subsection *Strobus* had high support, and *P. bhutanica* was most closely related to *P. wangii* Hu et Cheng (Figure 3).

## Discussion and conclusions

The chloroplast genome of *Pinus bhutanica* provides valuable information for understanding chloroplast genome evolution and phylogenetic inference in subsection *Strobus* in genus *Pinus*. In this study, the complete chloroplast genome of *P. bhutanica* was first reported and found to exhibit a total length of 116,919 bp. A total of 106 functional genes were annotated in the genome, including 72 PCGs, 30 tRNA genes, and four rRNA genes. The genome size and gene content of *Pinus bhutanica* is not significantly different from those of most chloroplast genomes in the subsection *Strobus* (Zeb et al. 2020), indicating that the genome evolution was relatively conservative in the subsection. The phylogenetic analysis revealed that *P. bhutanica* is most closely related to *Pinus wangii* in subsection *Strobus*. However, the discordance among the phylogenies from different publications was found for *P. bhutanica* and some other taxa in the subsection (Duan et al. 2016; Yang et al. 2018; Baturina et al. 2019; Schott et al. 2019; Zeb et al. 2019, 2020), suggesting that incomplete lineage sorting of chloroplast genome and/or introgressive hybridization among species might occur in the subsection. Therefore, more complete chloroplast genomes from subsection *Strobus* are necessary for further research on phylogenetic relationships in the subsection.

## Supplementary Material

Supplemental Material

Supplemental Material

## Data Availability

The genome sequence data that support the findings of this study are openly available in the GenBank of NCBI (https://www.ncbi.nlm.nih.gov/) under the accession number OP747469. The associated BioProject, SRA, and Bio-Sample numbers are PRJNA898140, SRR22188315, and SAMN31601598, respectively. A specimen of *Pinus bhutanica* was deposited at Jiangxi Agricultural University (https://www.jxau.edu.cn; contact person and email: Jing Wang, wangjing_genomics@163.com) under the voucher number 2021-PB-1A.
