# Supplementary material for: The complete chloroplast genome sequence of Pinus bhutanica (Pinaceae) and its phylogenetic implications
Source: Mitochondrial DNA B Resour. 2024 Jan 26;9(1):182–5. doi: 10.1080/23802359.2024.2305710 (PMC10823889; doi:10.1080/23802359.2024.2305710)
Supplement: Supplemental Material [file TMDN_A_2305710_SM2896.pdf]

Supplemental material

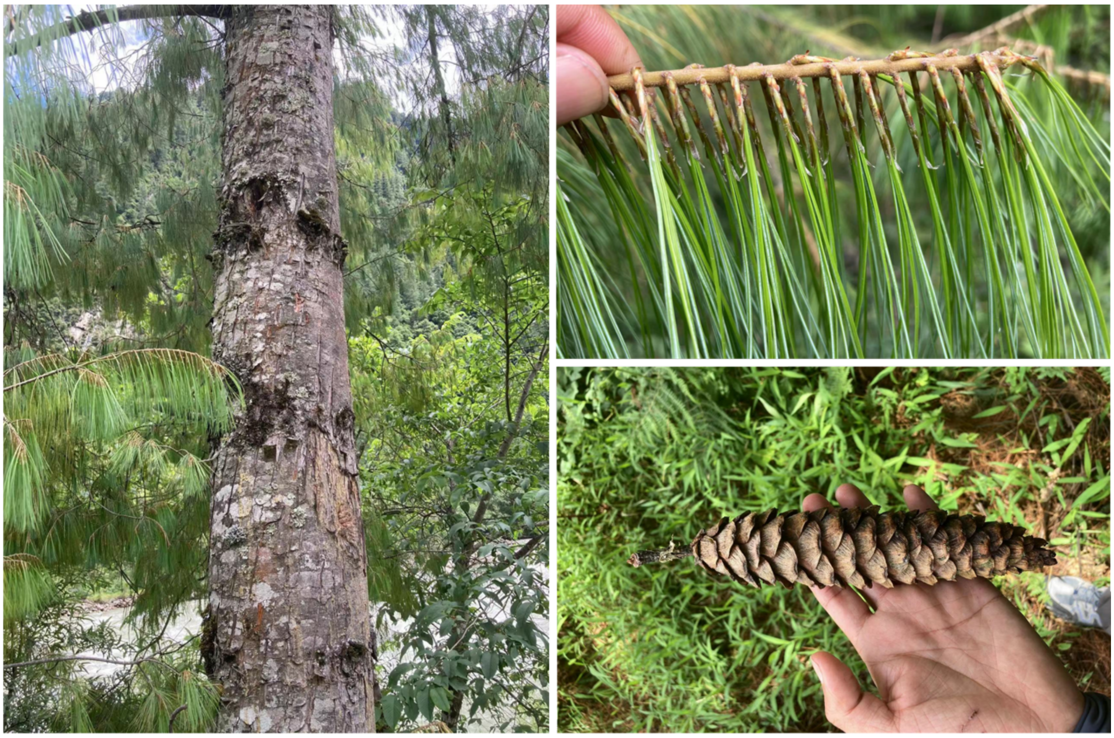

Figure S1. The images for the detailed phenotypes of *Pinus bhutanica* living in Linzhi, Xizang, China (geographic coordinates: 29º13'27" N, 95º11'3" E; photography by Yixuan Kou and Jing Wang).

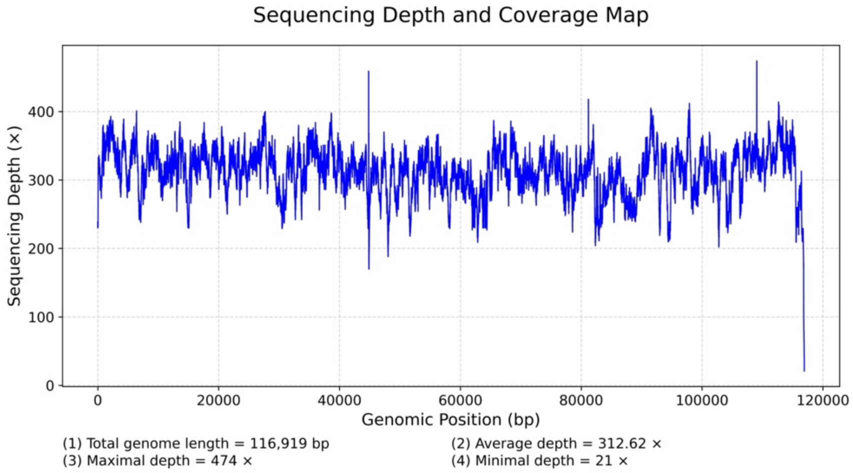

Figure S2. Read coverage depth of *Pinus bhutanica* chloroplast genome assembly. This figure was generated using a protocol which is available at https://www.protocols.io/view/generating-sequencing-depth-and-coverage-map-for-o-4r3l27jkxg1y/v1.

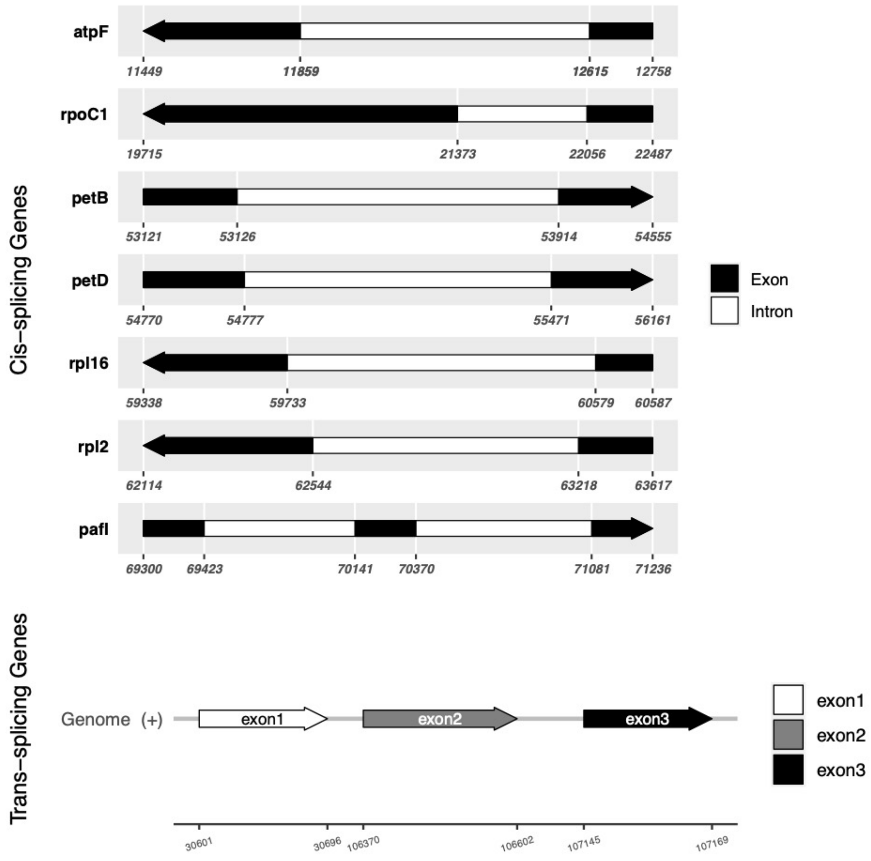

Figure S3. Schematic map of the cis-splicing genes and trans-splicing gene (*rps12*) in the *Pinus bhutanica* chloroplast genome. Arrows indicate the sense direction of genes. The map was generated using CPGview.
