# Supplementary material for: The complete chloroplast genome sequence of Pinus bhutanica (Pinaceae) and its phylogenetic implications
Source: Mitochondrial DNA B Resour. 2024 Jan 26;9(1):182–5. doi: 10.1080/23802359.2024.2305710 (PMC10823889; doi:10.1080/23802359.2024.2305710)
Supplement: Supplemental Material [file TMDN_A_2305710_SM2895.pdf]

SPRINGER NATURE
Author Services

Editing Certificate

This document certifies that the manuscript

The complete chloroplast genome sequence of Pinus bhutanica (Pinaceae) and its phylogenetic implications

prepared by the authors

Shiqi Lv, Jiao Chen, Bingbing Li, Taotao Fu, Mingliang Song, Pengtao Zhang, Kang Liu, Yixuan Kou and Jing Wang

was edited for proper English language, grammar, punctuation, spelling, and overall style by one or more of the highly qualified native English speaking editors at SNAS.

This certificate was issued on **July 10, 2023** and may be verified on the SNAS website using the verification code **413C-AE58-2BBF-F1D5-6A80**.

Neither the research content nor the authors' intentions were altered in any way during the editing process. Documents receiving this certification should be English-ready for publication; however, the author has the ability to accept or reject our suggestions and changes. To verify the final SNAS edited version, please visit our verification page at secure.authorservices.springernature.com/certificate/verify.
If you have any questions or concerns about this edited document, please contact SNAS at support@as.springernature.com.

SNAS provides a range of editing, translation, and manuscript services for researchers and publishers around the world.
For more information about our company, services, and partner discounts, please visit authorservices.springernature.com.
